# Experimental verification of position sensing for a magnetic coil via Fano resonance

**DOI:** 10.1038/s41598-025-02989-7

**Published:** 2025-07-01

**Authors:** Shrinathan Esaki Muthu Pandara Kone, Kenichi Yatsugi, Mikiko Suzuki, Michio Yasunishi, Hiroshi Yoshimoto, Xiaopeng Li, Ziqi Yu, Taehwa Lee, Hideo Iizuka

**Affiliations:** 1 Toyota Central R&D Labs, Nagakute, Aichi 480 1192 Japan; 2 Toyota Motor Corporation, Toyota, Aichi 471 8571 Japan; 3 Toyota Research Institute of North America, Toyota Motor North America, Ann Arbor, MI 48105 USA

**Keywords:** Magnetic coupling, Magnetic resonance, Fano resonance, Coils, Machine learning, Applied physics, Electronics, photonics and device physics

## Abstract

Predicting the position of a magnetic noise source in the kilohertz/megahertz range is of interest in academia and importance in industries. We consider a system consisting of one transmitting and four receiving coils around a frequency of 10 MHz. The transmitting coil is assumed to be a magnetic noise source that needs to be found in our study. We theoretically and experimentally show that the position of the transmitting coil can be predicted using Fano resonance, i.e., the four receiving coils are strongly coupled with each other, and weakly coupled to the transmitting coil via magnetic fields, by employing supervised machine learning. A coupled mode theory is built to elucidate such magnetic resonance coupling among coils. The characteristics of the system are investigated by using a method-of-moment based electromagnetic simulator, with sufficiently large volume of scattering parameters for different positions of the transmitting coil. Measured spectra of scattering parameters reasonably agree with analytical and numerical results. Experimental results reveal that the position of the transmitting coil is predicted in a range of 0.4 m to 2 m for the distance and ± 60º for the angle, with resolutions of 0.048 m and 8.8º, respectively.

Predicting the position of a source emitting waves or direction of an incoming wave in a limited device size is of fundamental interest in academia and importance in industries. For the far-field regime, it is well known that a receiver consisting of two elements at least needs a half-wavelength size when received wave-phases are compared to predict the direction of an incoming wave. Inspired from the mechanism of detecting the direction of sound waves in small creatures with their sizes much less than the sound wavelengths, the comparison of received wave-amplitudes at two elements allowed subwavelength receivers, which were experimentally verified in optics^1^, microwaves^2^, and acoustics^3^. The two elements were strongly wave-coupled in those receivers, generating interference between direct and resonance-assisted indirect pathways, which is called Fano resonance^4–7^.

In the kilohertz/megahertz range, coils are widely used as transmitting and receiving elements^8–11^. Predicting the position of a coil can improve the efficiency of wireless power transfer^12^. In addition, in electromagnetic compatibility, predicting the positions of magnetic noise sources is desired for efficient development process in industries^13–16^. Recently, a theoretical work in Ref.^17^ has reported that the position of a transmitting coil can be predicted by a simple system consisting of two receiving coils, which needs to be mechanically rotated. The mechanical rotation provides the ratio of two received wave-amplitudes in a finite angular range. The ratio and its angular derivative lead to an analytical solution of the distance and angle of the transmitting coil. Increasing the number of receiving coils can eliminate the mechanical rotation of the receiving system. However, predicting the position of a coil is a challenge as the number of receiving coils increases. Moreover, the position sensing capability has not been experimentally verified.

As a step further, in this paper, we theoretically and experimentally investigate position sensing capability in a system consisting of one transmitting coil as a magnetic noise source, and four receiving coils around a frequency of 10 MHz. The four receiving coils are strongly coupled with each other and weakly coupled to the transmitting coil via magnetic fields. We show that the position of the transmitting coil can be predicted from wave-amplitudes in the four receiving coils. Machine learning is a powerful approach for solving complex challenges in various research fields^18–21^. Here, supervised machine learning is employed in our system to perform the prediction even when received wave-amplitudes for a position of the transmitting coil are very similar to those for other positions of it. Experimental results reveal that the position of the transmitting coil is predicted in a range of 0.4 m to 2 m for the distance and ± 60º for the angle, with resolutions of 0.048 m and 8.8º, respectively.

## Results

### Configuration of coil position sensing

**Fig. 1:**
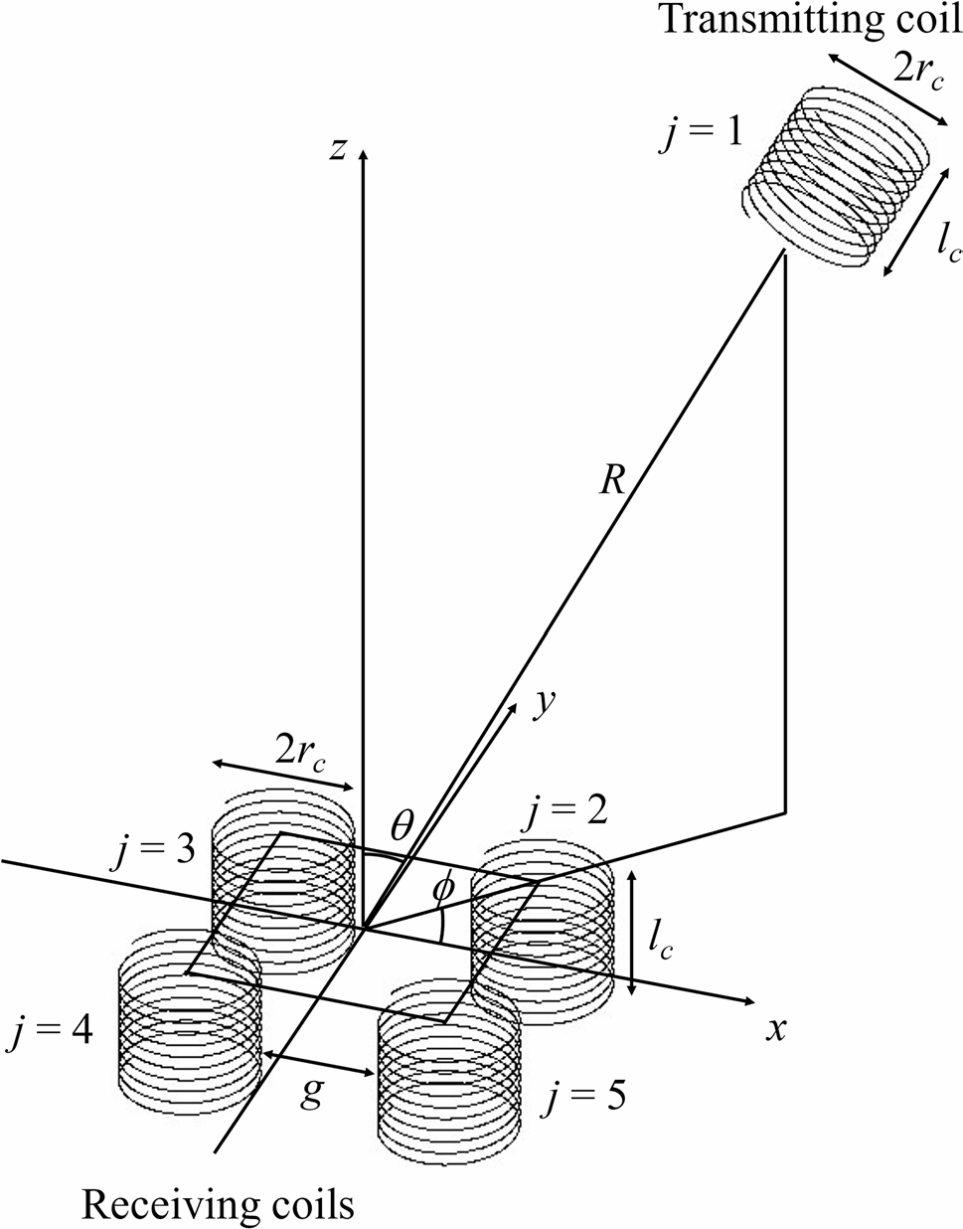
Configuration of a system consisting of one transmitting coil (*j* = 1) and four receiving coils (*j* = 2 to 5). The position of the transmitting coil is able to be predicted from wave-amplitudes in the four receiving coils using Fano resonance. Dimensions of the identical coils are presented in Table 1. A gap distance of *g* = 0.2 m between receiving coils is selected unless otherwise mentioned.

We consider a system consisting of one transmitting coil (*j* = 1) and four receiving coils (*j* = 2 to 5), as shown in Fig. 1. The transmitting coil is positioned at (*R*,*θ*,*φ*) in the spherical coordinate system with its axis directed to the origin of the coordinate system, where *R* is the distance from the origin to the nearest side of the transmitting coil, *θ* is the elevation angle from the *z* axis, and *φ* is the azimuth angle from the *x* axis in the *xy* plane. Note that the position of the transmitting coil is alternatively expressed by (*x*,*y*,*z*) = (*R*sin(*θ*)cos(*φ*),*R*sin(*θ*)sin(*φ*),*R*cos(*θ*)) in the Cartesian coordinate system. The receiver consists of identical four coils arranged in a square lattice with each coil axis being parallel to the *z* axis. The top of each coil lies in the *xy* plane. The *x* and *y* axes pass through the origin of the spherical coordinate system. The origin is located at the center of the square, where the four coils (*j* = 2 to 5) are placed at its four vortices.

Our sensing scheme has a feature to select a narrow gap distance of *g* = 0.2 m among the receiving coils for strong coupling, and distance *R* between the transmitting coil and the receiver is varied from 0.4 m to 2 m for weak coupling. This combination of strong and weak coupling enables the effective use of the four receiving coils for predicting a position of the transmitting coil, i.e., excited magnetic fields in the four coils are largely varied. For example, suppose the transmitting coil is positioned in the region of *x* > 0 and *y* > 0. In our sensing scheme, the furthest coil (*j* = 4) can have the strongest magnetic field excitation due to the interference of magnetic fields among four coils, depending on the position of the transmitting coil. In other words, four coils can effectively contribute to the position sensing performance. On the other hand, a conventional sensing scheme has weak coupling among four coils, i.e., large distance *g* in the configuration of Fig. 1. The nearest coil (*j* = 2) always has the strongest magnetic field excitation and the dominant contribution to the position sensing performance, with the other coils (*j* = 3, 4, and 5) having small contribution to it. These behaviors will be numerically compared later. In addition to the sensing performance, our sensing scheme with narrow gap distance *g* has an advantage of small size, comparing with the conventional one with large *g*.

The parameters of the identical coils are presented in Table 1. Fabricated coils, which will be shown later, and the numerically modeled coils of Fig. 1 have the same dimensions of coil radius *r*_*c*_ = 80 mm and coil length *l*_*c*_ = 153 mm. The fabricated coil consists of a metallic strip with a *w*_*s*_ = 2 mm width, and has a number of wire turns of *N*_*w*_ = 25, showing a resonance frequency of *f*_0_ = 9.79 MHz. In the numerically modeled coil consisting of a cylindrical wire, the number of wire turns and wire diameter are adjusted to *N*_*w*_ = 25.2 and *d*_*w*_ = 3.8 mm, respectively, so that the resonance frequency of the numerically modeled coil coincides with *f*_0_ = 9.79 MHz of the fabricated coil.

**Table 1:** Dimensions of identical coils. Each coil consists of a cylindrical wire in the numerical model and a copper strip in the experimental setup. The parentheses correspond to the experimental setup.

Parameters	Values
Coil radius *r*_*c*_	80 mm
Coil length *l*_*c*_	153 mm
Number of wire turns *N*_*w*_	25.2 (25)
Wire diameter *d*_*w*_ (Strip width *w*_*s*_)	3.8 mm (2 mm)

### Coupled mode theory

**Fig. 2:**
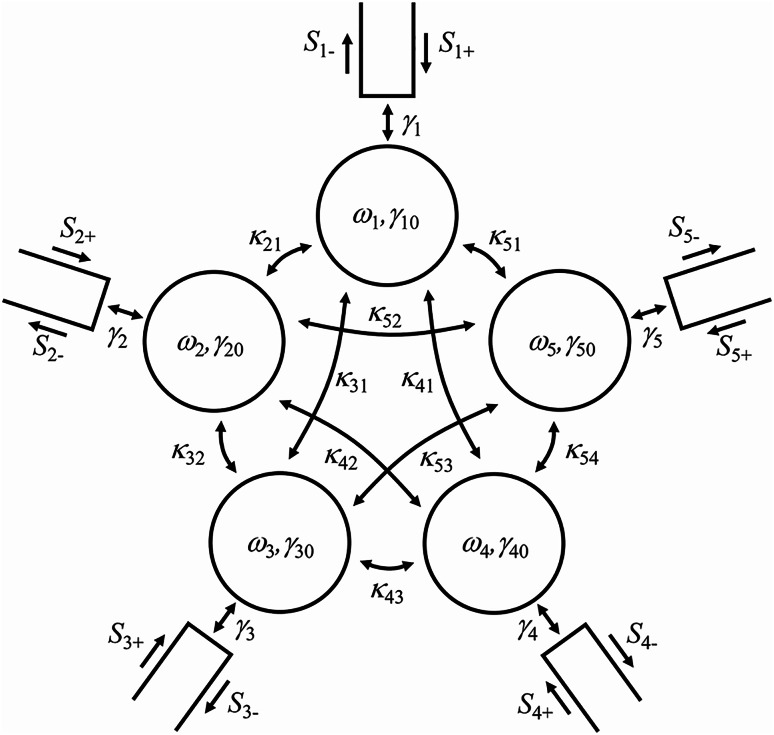
Analytical model of the system in Fig. 1 for the coupled mode theory.

Magnetic resonance coupling phenomena in the kilohertz/megahertz range have been investigated in detail in the wireless power transfer technology by using the circuit theory^9–12^ and coupled mode theory^8,10^. The coupled mode theory can describe how plural resonators are coupled in a simple manner and provide a clear picture of a complex coupling phenomenon. In Ref.^17^, the coupled mode theory revealed a unique magnetic coupling phenomenon among one transmitting and two receiving coils in Fano resonance, where the far-side receiving coil has stronger magnetic field excitation than the near-side receiving coil. Here, we extend it to the case of four receiving coils with a transmitting coil. We assume that the system is reciprocal, and the far-field radiation is negligibly small. Figure 2 shows the analytical model of the system of Fig. 1. The analytical model consists of five resonators coupled with each other. Each resonator couples to the corresponding port. We assume the *e*^*iωt*^ convention, where *ω* and *t* are the angular frequency and time. The system dynamics of the analytical model is given by^22,23^

1 $$\frac{d}{dt}\varvec{a}=\widehat{A}\varvec{a}+\widehat{B}{\varvec{S}}_{+},$$

2 $${\varvec{S}}_{-}=-{\varvec{S}}_{+}+\widehat{B}\varvec{a},$$

where

3 $$\widehat{A}=\left(\begin{array}{ccc}{A}_{11}&\:i{\kappa\:}_{21}&\:\begin{array}{ccc}i{\kappa\:}_{31}&\:i{\kappa\:}_{41}&\:i{\kappa\:}_{51}\end{array}\\\:i{\kappa\:}_{21}&\:{A}_{22}&\:\begin{array}{ccc}i{\kappa\:}_{32}&\:i{\kappa\:}_{42}&\:i{\kappa\:}_{52}\end{array}\\\:\begin{array}{c}i{\kappa\:}_{31}\\\:i{\kappa\:}_{41}\\\:i{\kappa\:}_{51}\end{array}&\:\begin{array}{c}i{\kappa\:}_{32}\\\:i{\kappa\:}_{42}\\\:i{\kappa\:}_{52}\end{array}&\:\begin{array}{ccc}{A}_{33}&\:i{\kappa\:}_{43}&\:i{\kappa\:}_{53}\\\:i{\kappa\:}_{43}&\:{A}_{44}&\:i{\kappa\:}_{54}\\\:i{\kappa\:}_{53}&\:i{\kappa\:}_{54}&\:{A}_{55}\end{array}\end{array}\right),$$

4 $${A}_{jj}=i{\omega\:}_{j}-{\gamma\:}_{0j}-{\gamma\:}_{j},$$

5 $$\widehat{B}=diag\left(\sqrt{2{\gamma\:}_{1}}\:\:\:\sqrt{2{\gamma\:}_{2}}\:\:\:\sqrt{2{\gamma\:}_{3}}\:\:\:\sqrt{2{\gamma\:}_{4}}\:\:\:\sqrt{2{\gamma\:}_{5}}\right),$$

6 $$\varvec{a}={\left({a}_{1}\:\:\:{a}_{2}\:\:\:{a}_{3}\:\:\:{a}_{4}\:\:\:{a}_{5}\right)}^{T},$$

7 $${\varvec{S}}_{+}={\left({S}_{1+}\:\:\:{S}_{2+}\:\:\:{S}_{3+}\:\:\:{S}_{4+}\:\:\:{S}_{5+}\right)}^{T},$$

8 $${\varvec{S}}_{-}={\left({S}_{1-}\:\:\:{S}_{2-}\:\:\:{S}_{3-}\:\:\:{S}_{4-}\:\:\:{S}_{5-}\right)}^{T},$$

*a*_*j*_ is the mode amplitude of resonator *j* and normalized so that |*a*_*j*_|^2^ represents the mode energy. *ω*_*j*_ and *γ*_0*j*_ are the resonance frequency and the intrinsic loss rate of resonator *j*. *κ*_*jl*_ is the coupling rate between resonators *l* and *j*, where *κ*_*jl*_ = *κ*_*lj*_ due to reciprocity. *S*_*j*+_ and *S*_*j*-_ are the input and output wave-amplitudes at port *j*. *γ*_*j*_ is the coupling rate between resonator *j* and port *j*. From (1) and (2), in the frequency domain, we have

9 $${\varvec{S}}_{-}=\left[-\widehat{I}+\widehat{B}{\left(i\omega\:\widehat{I}-\widehat{A}\right)}^{-1}\widehat{B}\right]{\varvec{S}}_{+},$$

where $\:\widehat{I}$ is the 5$\:\times\:$5 identity matrix, and the determinant of (*iω*$\:\widehat{I}-\widehat{A}$) is non-zero. In the case of identical coils with *ω*_*j*_ = *ω*_0_, *γ*_0*j*_ = *γ*_00_, and *γ*_*j*_ = *γ*_0_, i.e., *A*_*jj*_ = *A*_00_, for *j* = 1 to 5, Eq. (9) is simplified

10 $${\varvec{S}}_{-}=\left[-\widehat{I}+2{\gamma\:}_{0}{\left(i\omega\:\widehat{I}-\widehat{A}\right)}^{-1}\right]{\varvec{S}}_{+} =\widehat{M}\left(R,\theta\:,\varphi\:\right){\varvec{S}}_{+},$$

where matrix $\:\widehat{M}$ is dependent on position (*R*,*θ*,*φ*) of the transmitting coil since coupling rates among resonators are dependent on the position, i.e., $\:\widehat{A}=\widehat{A}$(*R*,*θ*,*φ*). When an incident wave is excited at port 1 with *S*_1+_ = 1 and *S*_2+_ = *S*_3+_ = *S*_4+_ = *S*_5+_ = 0, the output waves *S*_2-_, *S*_3-_, *S*_4-_, and *S*_5-_ at ports 2 to 5 are calculated from Eq. (10), which is written as

11 $$\frac{{S}_{j-}}{{S}_{1+}}\equiv\:{S}_{j1}={\widehat{M}}_{j1}\left(R,\theta\:,\varphi\:\right),\:\:\:j=2\:to\:5 .$$

Therefore, position (*R*,*θ*,*φ*) of the transmitting coil can, in principle, be analytically determined using four receiving coils if (*R*,*θ*,*φ*) is uniquely linked to four scattering parameters set (*S*_21_,*S*_31_,*S*_41_,*S*_51_), which is alternatively given by the normalized form of (*S*_31_/*S*_21_,*S*_41_/*S*_21_,*S*_51_/*S*_21_) with *S*_21_ ≠ 0.

### Numerical characteristics of the coil system

**Fig. 3:**
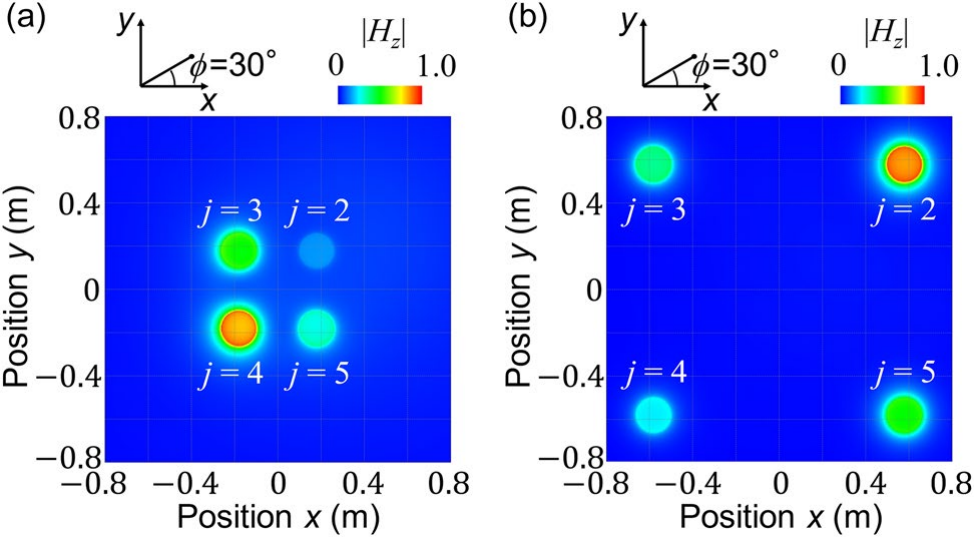
Distributions of the *z*-component magnetic field |*H*_*z*_| in the four receiving coils of Fig. 1 at *f*_0_ = 9.79 MHz in the *xy* plane (*z* = -*l*_*c*_/2), which are obtained by using Altair FEKO. The transmitting coil is positioned at *R* = 1 m, *θ* = 30º, and *φ* = 30º, corresponding to the top right direction in the figure, and is not presented in the figure. The gap distances between receiving coils are selected at (**a**) *g* = 0.2 m (proposed) and (**b**) *g* = 1 m (conventional), respectively. Fano resonance occurs in (**a**), where the furthest coil (*j* = 4) has the strongest magnetic field excitation.

The system of Fig. 1 is numerically investigated by using the method-of-moment based electromagnetic simulator Altair FEKO^24^. Figure 3a shows the magnetic field distribution of the four receiving coils in the *xy* plane (*z* = -*l*_*c*_/2) at *f*_0_ = 9.79 MHz when a gap distance of *g* = 0.2 m among the four receiving coils is selected. The transmitting coil is positioned at *R* = 1 m, *θ* = 30º, and *φ* = 30º, i.e., *x* = *R*sin(*θ*)cos(*φ*) > 0 and *y* = *R*sin(*θ*)sin(*φ*) > 0 (top right direction in Fig. 3). We observe that the furthest coil (*j* = 4) has the strongest magnetic field excitation, and the nearest coil (*j* = 2) has the weakest magnetic field excitation, which is the unique feature of Fano resonance arising from the interference of strong coupling among the receiving coils and weak coupling between each receiving coil and the transmitting coil. As gap distance *g* is wider, magnetic coupling among the four coils reduces. Figure 3(b) shows the magnetic field distribution for *g* = 1 m. The nearest coil (*j* = 2) has the strongest magnetic field excitation, as can be seen in other typical resonance phenomena, limiting the position sensing performance via the dominant contribution of a single coil.

**Fig. 4:**
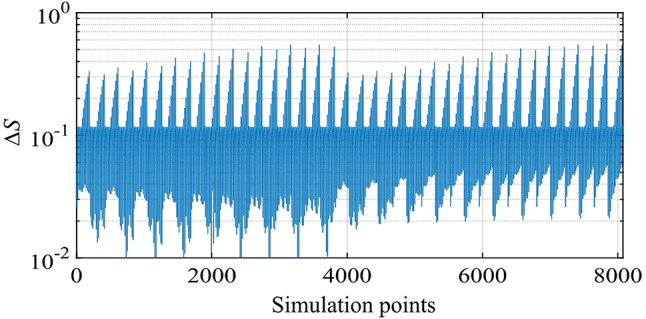
Minimum distance among |S_21_|, |S_31_|, |S_41_|, and |S_51_| at *f*_0_ = 9.79 MHz, which is defined in Eq. (12), for each of all data points *N* = 8075. The position of the transmitting coil is varied, as presented in Table 2.

Next, we clarify the relation between the position of the transmitting coil and wave-amplitudes in the four receiving coils through scattering parameters. The position of the transmitting coil is varied in the range of 0.4 m ≦ *R* ≦ 2 m, -60º ≦ *θ* ≦ 60º, and − 45º ≦  φ≦ 45º, as presented in Table 2, considering symmetry of the structure in Fig. 1, with *N* = 8075 (= 17 × 25 × 19 for *R*, *θ*, and *φ*) data points. Scattering parameters *S*_21_, *S*_31_, *S*_41_, and *S*_51_ are numerically obtained at *f*_0_ = 9.79 MHz. To understand the characteristics of those scattering parameters, the minimum distance among the four scattering parameters for each of all data points *N* = 8075 is analyzed. The minimum distance of the *m*th data point is defined as

12 $${\varDelta\:S}_{m}=\underset{\begin{array}{c}n=1\:to\:N,\\\:n\ne\:m\end{array}}{\text{m}\text{i}\text{n}}\sqrt{\sum\:_{j=2}^{5}{\left(\left|{S}_{m,j1}\right|-\left|{S}_{n,j1}\right|\right)}^{2}},$$

and is plotted in Fig. 4. Some data points have relatively large *ΔS* > 0.1, where the scattering parameter set of the *m*th data point is uniquely distinguished. However, other data points have small *ΔS*, i.e., the scattering parameter set of the *m*th data point is very similar to, at least, one of the other data points. Such small *ΔS* comes from two reasons. One is that there are regions where our system hardly determines the position of the transmitting coil. The other is that the step of the parameter change is less than the resolution of the sensing performance. In the four receiving-coil system with small *ΔS* (insufficient differentiability), uniquely predicting the position of the transmitting coil becomes computationally complex using the analytical model of Fig. 2, which would be even more challenging as the number of coils increases. Machine learning is employed to tackle such complex phenomena.

**Table 2:** Variation range of the position of the transmitting coil in the numerical model.

Parameters	Values
Distance	0.4 m ≦ *R* ≦ 2 m with a step of 0.1 m
Elevation angle	− 60º ≦ *θ* ≦ 60º with a step of 5º
Azimuth angle	− 45º ≦ *φ*≦ 45º with a step of 5º

### Experimental characteristics of the coil system

**Fig. 5:**
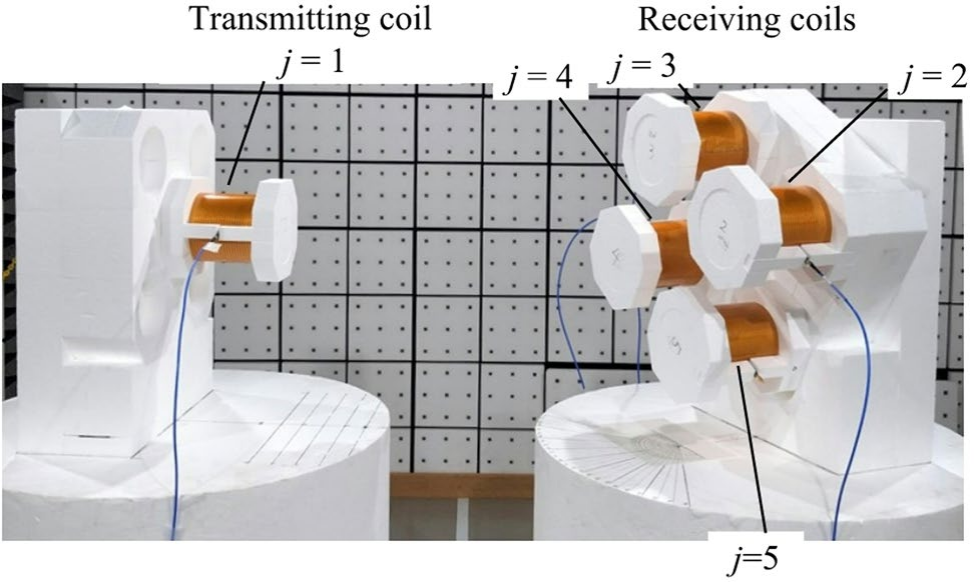
Experimental setup of the coil position sensing system in Fig. 1.

Based on the numerical results above, here, the magnetic coupling among the coils is experimentally validated in an anechoic chamber. Figure 5 shows the experimental setup of the system consisting of one transmitting coil and four receiving coils. Five fabricated coils were mechanically supported by styrene foams. The position of the transmitting coil was effectively varied in two ways in Table 3, i.e., one was the “wide range”, where *R* and *θ* were sparsely varied in the same range as the numerical calculation in Table 2, and *φ* was selected at 0º, 30º, and 45º as a proof-of-concept investigation. The other was the “fine step”, where one parameter *R* or *θ* was varied with a fine step and the other two parameters were fixed.

**Table 3:** Variation range of the position of the transmitting coil in the experimental setup.

	Parameters	Values
Wide range	Distance	*R* = 0.5 m, 1 m, 2 m
	Elevation angle	− 60º ≦ *θ* ≦ 60º with a step of 15º
	Azimuth angle	*φ* = 0º, 30º, 45º
Fine step	Distance	0.4 m ≦ *R* ≦ 2 m with a step of 0.1 m, *θ* = 30º and *φ* = 30º fixed
	Elevation angle	− 60º ≦ *θ* ≦ 60º with a step of 5º, *R* = 1 m and *φ* = 30º fixed

**Fig. 6:**
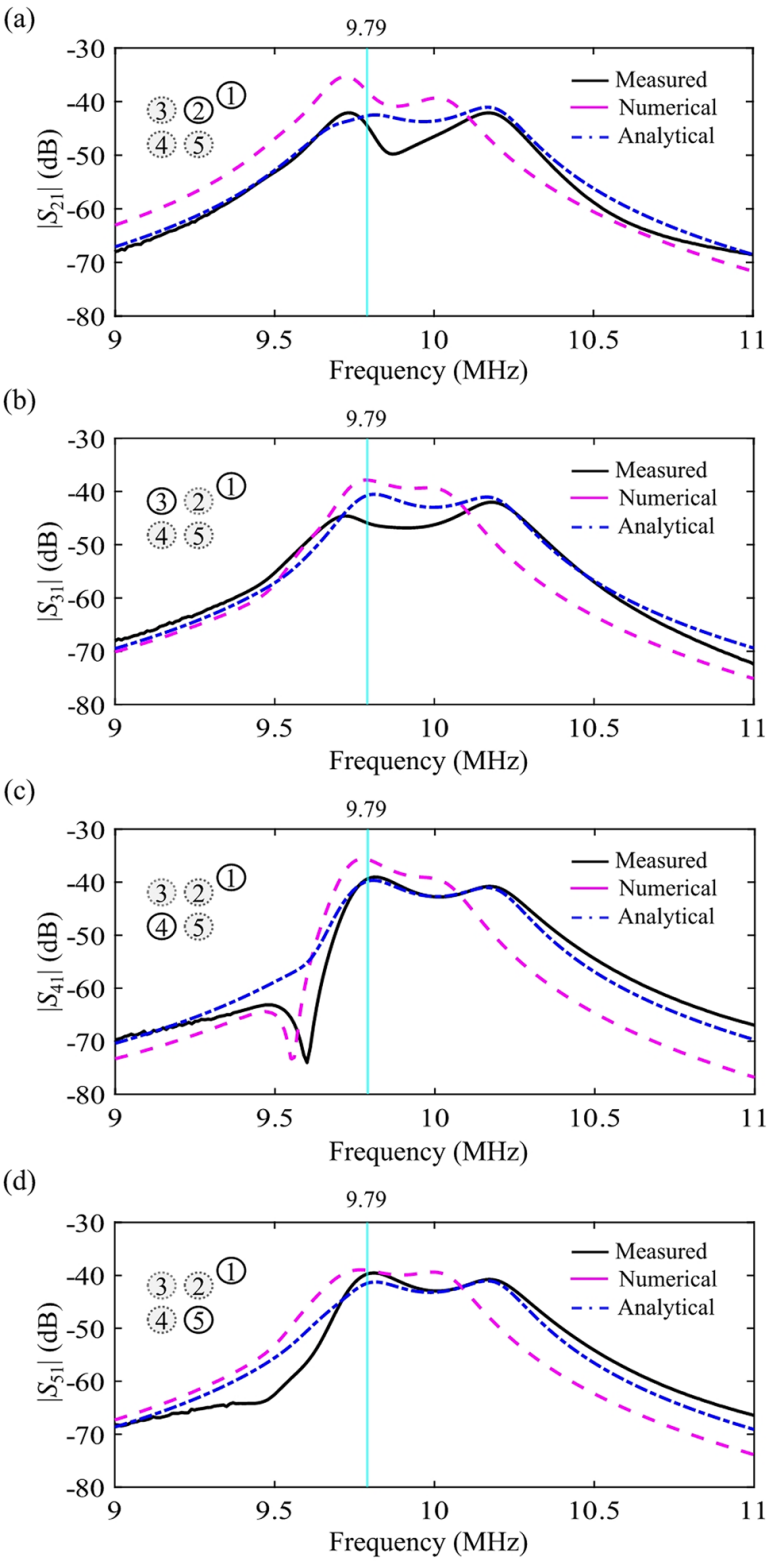
Measured spectra of (**a**) |*S*_21_|, (**b**) |*S*_31_|, (**c**) |*S*_41_|, and (**d**) |*S*_51_| for the system of Fig. 5. (*R* = 1 m, *θ* = 30º, and *φ* = 30º, black solid lines: measured, pink dashed lines: Altair FEKO, blue dashed-dotted lines: coupled mode theory Eq. (11) with parameters of *ω*_0_/(2π) = 9.79 MHz, (*γ*_0_ + *γ*_00_)/(2π) = 0.097 MHz, *κ*_21_/(2π) = 2 kHz, *κ*_31_/(2π) = 1.7 kHz, *κ*_41_/(2π) = 1.6 kHz, *κ*_51_/(2π) = 1.8 kHz, *κ*_32_/(2π) = *κ*_43_/(2π) = *κ*_54_/(2π) = *κ*_52_/(2π) = 0.15 MHz, and *κ*_42_/(2π) = *κ*_53_/(2π) = 0.1 MHz).

Typical measured spectra of scattering parameters |*S*_21_|, |*S*_31_|, |*S*_41_|, and |*S*_51_| are shown in Fig. 6a-d, respectively. Ripples are observed around the resonance frequency of *f*_0_ = 9.79 MHz, which occur in each receiving coil from the interference of strong coupling to other receiving coils and weak coupling to the transmitting coil. The measured results (black solid lines) reasonably agree with numerical results (pink dashed lines) obtained by Altair FEKO and analytical results (blue dashed-dotted lines) based on the coupled mode theory Eq. (11) with the fitting parameters in the caption of Fig. 6.

Discrepancies among three curves are discussed. Firstly, we have checked that for the two-coil system, the prototype, numerical model, and analytical model have the same resonance frequency of *f*_0_ = 9.79 MHz. For the five-coil system in our study, the experimental results (black solid lines) have the blue shift and the two peaks split more widely, comparing with the numerical results (pink dashed lines). The analytical results (blue dashed-dotted lines) have the blue shift and the wide split of the two peaks as well by using the fitting parameters. Consequently, the discrepancies of the measured spectra from the numerical spectra dominantly arise from different coupling strengths, i.e., coupling among the coils for the numerical model, consisting of copper cylindrical wires, is weaker than that for the prototype, consisting of copper strips. One can check that the spectra of the analytical model would get close to the numerical results if smaller coupling rates are selected.

### Evaluation of sensing performance using machine learning

**Fig. 7:**
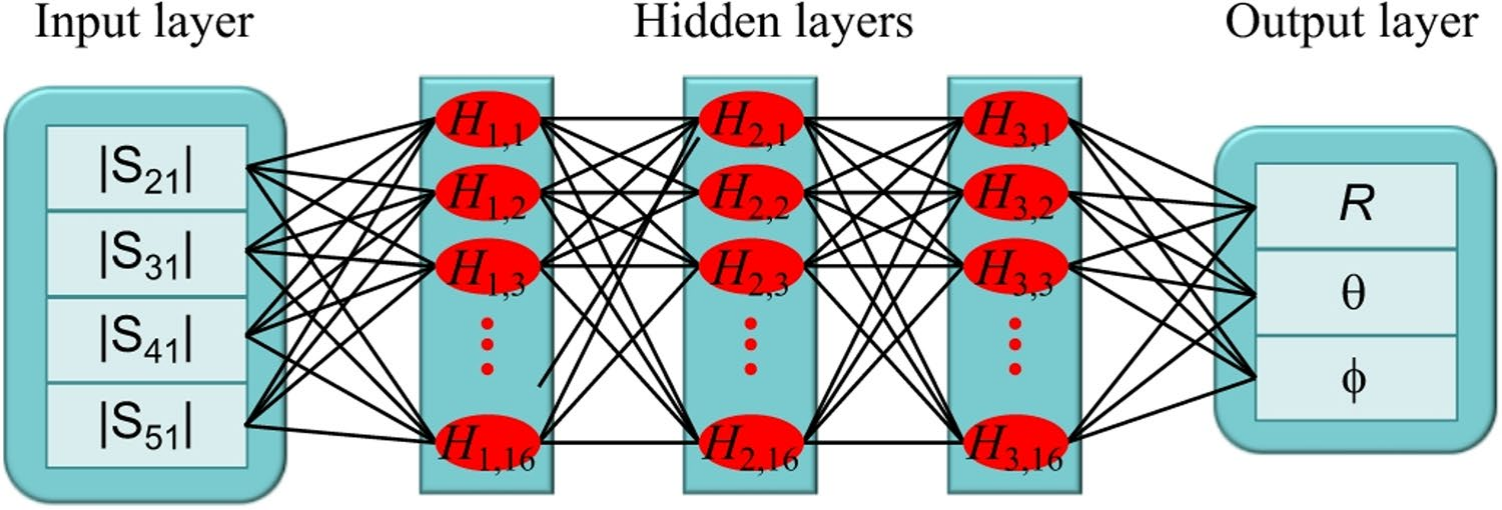
Architecture of machine learning for predicting the position of the transmitting coil.

Based on the numerical and experimental investigations, the position of the transmitting coil is predicted. We employ a supervised machine learning algorithm^25^. Figure 7 shows the architecture of the deep learning model, consisting of the input layer, 3 hidden layers with each having 16 neurons, and output layer. Each neuron in the hidden layers nearest to the input layer and output layer connects to each of scattering parameters |*S*_21_|, |*S*_31_|, |*S*_41_|, and |*S*_51_| in the input layer and position (*R*,*θ*,*φ*) of the transmission coil in the output layer, respectively. We use the Broyden-Fletcher-Goldfarb-Shanno (BFGS) optimization algorithm and the hyperparameters are presented in Table 4. Machine learning is carried out for numerical and experimental results, respectively, with *N* = 8075 numerical and *N* = 123 experimental data sets, where all scattering parameters are calculated and measured at *f*_0_ = 9.79 MHz. Numerical data sets of 5653 and 2422 and experimental data sets of 86 and 37 are assigned for training and testing, which correspond to a ratio of 7:3.

**Table 4:** Hyperparameters of the Broyden–Fletcher–Goldfarb–Shanno (BFGS) optimization algorithm.

Parameters	Values
Gradient tolerance	0.0001
Number of hidden layers	3
Number of neurons per layer	16
Epoch	2000

**Fig. 8:**
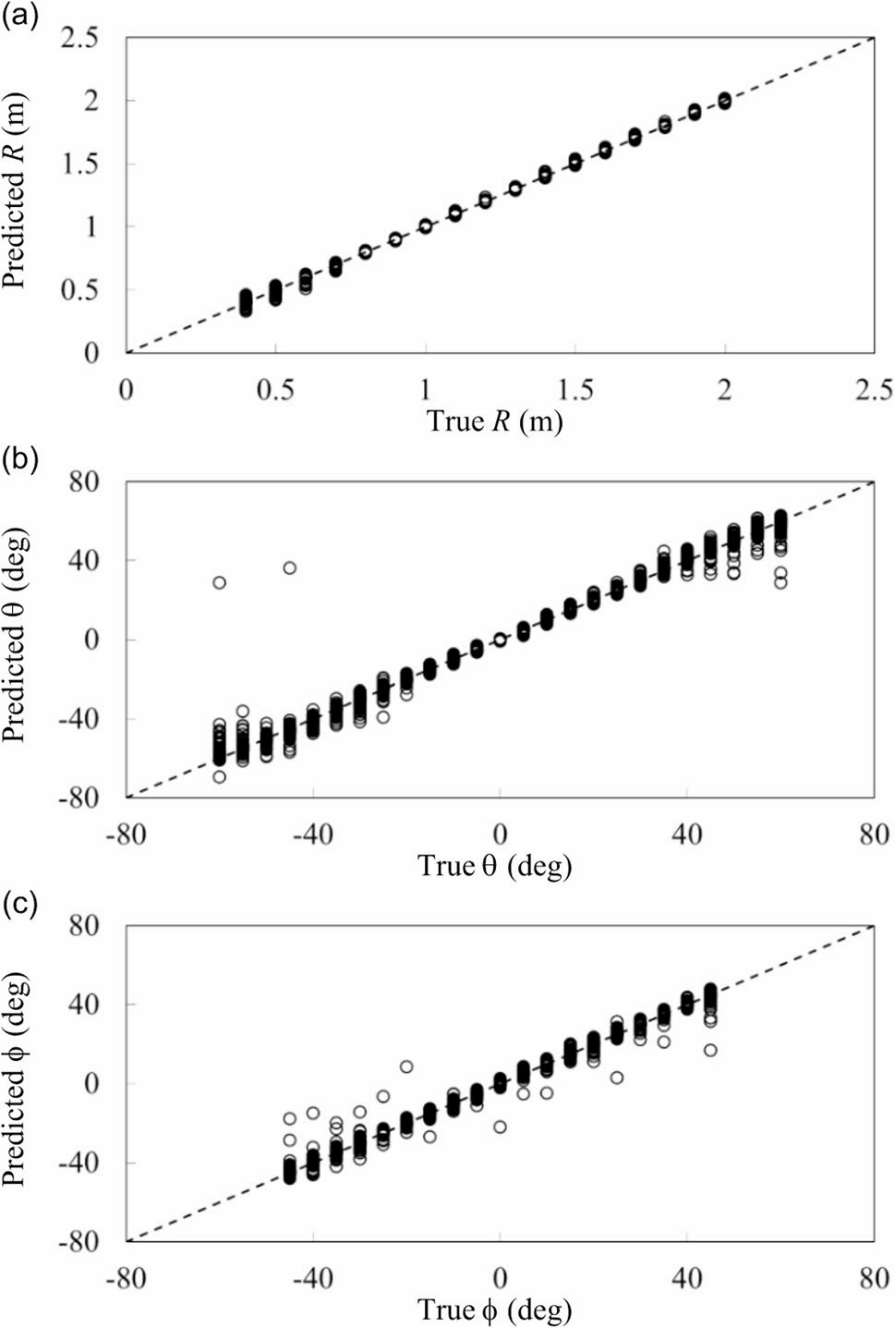
Predicted position of the transmitting coil from numerical scattering parameters (*N* = 8075). (**a**) Distance *R*, (**b**) elevation angle *θ*, and (**c**) azimuth angle *φ*.

Figure 8a–c show the prediction performance of distance *R*, elevation angle *θ*, and azimuth angle *φ* obtained from the *N* = 8075 numerical data set, and the resolutions (RMSE; root-mean square errors) are presented in Table 5. We see the excellent prediction for distance *R* and good prediction for angles *θ* and *φ*, with RMSE of 0.01 m, 3.4º and 2º, respectively.

**Fig. 9:**
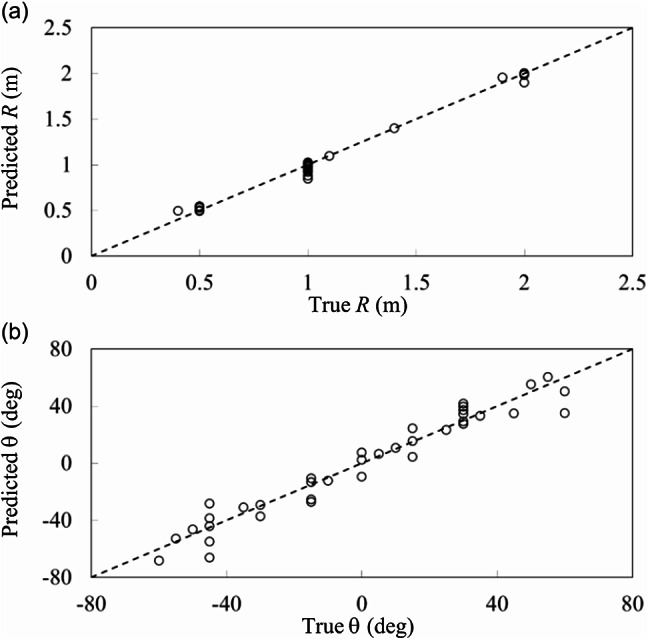
Predicted position of the transmitting coil from experimental scattering parameters (*N* = 123). (**a**) Distance *R* and (**b**) elevation angle *θ*.

Likewise, the prediction of distance *R* and elevation angle *θ* is performed from the *N* = 123 experimental data set, and the prediction performance is shown in Fig. 9a and b, with RMSE presented in Table 5. The good prediction for *R* and reasonable prediction for *θ* are obtained with RMSE of 0.048 m and 8.8º, respectively, in our sensing scheme even when the data set is relatively small. Note that *φ* was only varied at 0º, 30º, and 45º in the experiment, which is not sufficient for the prediction of *φ*. It would be interesting to explore if machine learning is able to even predict the *φ* angle unseen in the experiment, which corresponds to an extended position sensing capability of our physical system. Such benefit has been reported in a recent demonstration in acoustic directional sensing^21^.

From the comparison of numerical and experimental results in Table 5, the discrepancies of the experimental results from the numerical results may come from small number of the experimental data set.

**Table 5:** Resolution of the predicted position of the transmitting coil obtained from *N* = 8075 numerical and *N* = 123 experimental data sets.

	Parameters	Resolution (RMSE)
Numerical results	Distance *R*	0.01 m
	Elevation angle *θ*	3.4º
	Azimuth angle *φ*	2º
Experimental results	Distance *R*	0.048 m
	Elevation angle *θ*	8.8º
	Azimuth angle *φ*	–

## Discussion

We have used *N* = 8075 numerical and *N* = 123 experimental data sets for predicting the position of the transmitting coil. Here, we further investigate the prediction performance of our sensing scheme using some of the data sets.

Selecting data sets with *φ* = 30º fixed, i.e., *N* = 425 (= 17 × 25 for *R* and *θ*) numerical and *N* = 69 experimental data sets, the prediction of the position of the transmitting coil with variation of *R* and *θ* is performed using the deep learning model of Fig. 7, and the results are presented in Table 6. Numerical data sets of 298 and 127 and experimental data sets of 48 and 21 are assigned for training and testing. Comparing with Table 5, selecting the data set (*N* = 425) with *φ* = 30º improves RMSE for *θ* from 3.4º to 1.1º and degrades RMSE for *R* from 0.01 m to 0.018 m for numerical results, which may come from more similar data points for *θ* and reduced data points for *R*. For experimental results (*N* = 69), RMSE for *θ* and *R* slightly improve from 8.8º to 6º and 0.048 m to 0.036 m. Similarity in the data set may be pronounced although the number of the data points reduces.

**Table 6:** Resolution of the predicted position of the transmitting coil obtained from *N* = 425 numerical and *N* = 69 experimental data sets when the azimuth angle is fixed at *φ* = 30º.

	Parameters	Resolution (RMSE)
Numerical results	Distance *R*	0.018 m
	Elevation angle *θ*	1.1º
Experimental results	Distance *R*	0.036 m
	Elevation angle *θ*	6º

We have investigated the range of 0.4 m ≦ *R* ≦ 2 m, -60º ≦ *θ* ≦ 60º, and − 45º ≦ *φ* ≦ 45º in Table 2 for evaluating the performance of our sensing scheme, and our position sensing capability is not limited to the range. For example, *R* is varied from 2 m to 3 m with a step of 0.05 m, with *θ* = *φ* = 30º fixed, and then the machine learning calculation using *N* = 21 data points (16 and 5 for training and testing) gives a RMSE of 0.019 m for distance *R*.

Lastly, we compare the prediction performance of the system having a narrow gap distance of *g* = 0.2 m (proposed) and a wide gap distance of *g* = 1 m (conventional), respectively, which correspond to the effective contributions from the four coils (see in Fig. 3(a)) and the dominant contribution from a single coil (see in Fig. 3(b)) to the position sensing performance. *R* and *θ* are varied in the range of 0.4 m ≦ *R* ≦ 2 m with a step of 0.1 m and − 60º ≦ *θ* ≦ 60º with a step of 5º, as shown in Table 2, with *φ* = 30º fixed, i.e., *N* = 425 data set is used. The comparison of the numerical results in Table 7 ensures that the system having the narrow gap distance exhibits higher sensitivity (RMSE = 0.036 m) in distance *R*, comparing with the system having the wide gap distance (RMSE = 0.132 m).

**Table 7:** Comparison of the prediction performance of the system having gap distances of *g* = 0.2 m (proposed) and *g* = 1 m (conventional), respectively, which correspond to Fig. 3a and b. The variation range of *R* and *θ* in Table 2 is used with *φ* = 30º fixed.

Parameters	Resolution (RMSE)	
	g = 0.2 m (proposed)	g = 1 m (conventional)
Distance *R*	0.036 m	0.132 m
Elevation angle *θ*	6º	4.6º

In conclusion, we have investigated position sensing capability for a magnetic coil around 10 MHz through the use of Fano resonance. Employing supervised machine learning, experimental results revealed that the position of a transmitting coil was able to be predicted by four receiving coils, for a distance range of 0.4 m to 2 m and an angle range of ± 60º, with resolutions of 0.048 m and 8.8º, respectively. Numerical results from method-of-moment based simulations supported the experimental results. The coupled mode theory elucidated magnetic coupling among the coils. Our sensing scheme is applicable to scenarios where the operation frequency is known, e.g., detecting noise sources caused by power devices during switching operation, and improving the performance of wireless power transfer systems.

## Methods

Five coils in Fig. 1 were modeled by cylindrical wires with a diameter of *d*_*w*_ = 3.8 mm in Altair FEKO. A voltage of 1 V was applied at the port of the transmitting coil (*j* = 1), and scattering parameters were obtained from induced voltages at the ports of four receiving coils (*j* = 2 to 5), where all ports had a characteristic impedance of 50 Ω. Scattering parameters of the fabricated system in Fig. 5 were measured in an anechoic chamber by using Network Analyzer (E5071C, Keysight).

## Data Availability

The data that support the present study are available from the corresponding author upon reasonable request.
